# Supplementary material for: Experiences and perspectives on rapid-test diagnosis of tuberculosis, histoplasmosis and cryptococcosis in people with advanced HIV/AIDS disease in Porto Alegre, Brazil
Source: PLoS One. 2024 Nov 27;19(11):e0312204. doi: 10.1371/journal.pone.0312204 (PMC11602015; doi:10.1371/journal.pone.0312204)
Supplement: S1 Appendix — (DOCX) [file pone.0312204.s001.docx]

**APPENDIX S1**

**Semi-structured interview/focus group script**

**Guide topics for semi-structured interviews with patients.**

Open-ended presentation question, addressing how many years you have lived with HIV.

Theme 1 - Previous diagnostic experiences: Investigate the experiences of patients with a previous diagnosis of tuberculosis, histoplasmosis or cryptococcosis. What services were consulted? How long did it take to receive an accurate diagnosis? What were the challenges and obstacles faced during the process?

Theme 2 - Perceptions about the rapid diagnostic intervention: Inquire about patients' opinions regarding the rapid diagnostic intervention. What were your expectations regarding this method? What was your experience with rapid diagnosis like? How do they believe rapid diagnosis can impact their treatment journey and quality of life?

Theme 3 - Perceived benefits: Investigate the benefits that patients expect to obtain/have obtained with the rapid diagnostic intervention. Do they believe this could lead to a faster, more accurate diagnosis? How do they believe it can improve their health and quality of life? What are your hopes for this intervention?

Theme 4 - Barriers and Challenges: Explore potential barriers or challenges that patients anticipate facing during implementation of rapid diagnostic intervention. What are your concerns regarding accessibility, availability or costs? What obstacles have they experienced/believe that may impact the successful implementation of this intervention?

Theme 5 - Recommendations for Improvement: Solicit suggestions from patients on how rapid diagnostic intervention can be improved to better meet their needs and expectations. What additional features would they like to see available? What aspects of the diagnosis and treatment process do they believe can be improved?

**Guide themes for semi-structured interviews with managers.**

Open-ended presentation question, addressing how many years you have worked with HIV policies.

Theme 1 - Importance of rapid diagnostic intervention: Inquire about the perception of policymakers regarding the relevance of rapid diagnostic intervention in combating the diseases in question. How do they see the impact of this intervention on public health and health indicators?

Theme 2 - Experience with rapid diagnosis policies: Investigate the experience of policy makers in monitoring the implementation of the rapid diagnosis intervention. What are the existing policies and interventions? What are the main challenges faced in incorporating this approach into health policies?

Theme 3 - Development of policy flows: Explore how rapid diagnostic intervention can influence the development of policy flows related to the diagnosis and treatment of tuberculosis, histoplasmosis and cryptococcosis. What changes can be made to existing guidelines and protocols? Which points of attention/services should be engaged?

Theme 4 - Interfederative relationship: Investigate how rapid diagnostic intervention can impact the relationship between different levels of government (federal, state and municipal) in the implementation of health policies. What are the challenges and opportunities for intergovernmental cooperation? What strategies can be adopted to promote coordination and sharing of responsibilities?

Theme 5 - Barriers and challenges: Explore the possible barriers or challenges that managers anticipate facing during the implementation of the rapid diagnosis intervention. What are your concerns regarding accessibility, availability or costs? What obstacles do they believe could impact the successful implementation of this intervention? What additional features would they like to see available?

**Guide themes for focus groups with health professionals**

Open-ended presentation question, addressing how many years you have collaborated with people living with HIV.

Theme 1 - Perception of the importance of the intervention: Inquire about the professionals' perception regarding the relevance of rapid diagnostic intervention for the early and accurate diagnosis of these diseases in patients with advanced HIV. What were your earlier experiences? What are the main benefits they see in this approach?

Theme 2 - Experience with the intervention: Investigate the experience of professionals in using the rapid diagnosis intervention. What strategies do you use to communicate intervention with patients? What have the results been observed so far? What are the main challenges faced during implementation? What were the main lessons learned?

Theme 3 - Impact on clinical practice: Explore how the rapid diagnostic intervention affected the clinical practice of professionals. What changes have they observed in the detection and treatment of the diseases in question? How has this approach influenced clinical decision making? Was there any change in the relationship with patients or in the acceptability of the intervention by patients?

Theme 4 - Interdisciplinary collaboration: Investigate how the rapid diagnostic intervention promoted interdisciplinary collaboration between healthcare professionals. What are professionals' perceptions about the importance of collaboration between different health professionals for the success of the intervention? How does collaboration occur between health levels?

Theme 5 - Challenges and recommendations: Ask professionals about the main challenges they faced when using the rapid diagnosis intervention and their recommendations to improve its implementation. What are the main barriers faced? What are your suggestions for improving the effectiveness and efficiency of the intervention?
