# Supplementary material for: Experiences and perspectives on rapid-test diagnosis of tuberculosis, histoplasmosis and cryptococcosis in people with advanced HIV/AIDS disease in Porto Alegre, Brazil
Source: PLoS One. 2024 Nov 27;19(11):e0312204. doi: 10.1371/journal.pone.0312204 (PMC11602015; doi:10.1371/journal.pone.0312204)
Supplement: S3 Appendix — (DOCX) [file pone.0312204.s003.docx]

**Appendix S3**

**Summary table of professional groups**

**Focus Group 1**

| Profile | P1 - infectious disease specialist. I work at the hospital, and I have worked with people living with HIV since 2018, when I entered residency. | P2 - infectious disease specialist at Porto Alegre and has worked with HIV for over 20 years. | P3 - doctor, I work at the hospital, in the management area. I started treating patients with HIV in 2020, inside prisons. | P4 - r3 of clinical infection. Yeah, but I did internal medicine in clinics before, starting in 2019, and since then I've been working with people living with HIV. | P5 - medical student and the research started more or less in November, it was my first contact with an HIV patient. | P6 - medical student and it was with the beginning of this research, in November, that I had my first contact with patients living with HIV. | P7 - No information |
| --- | --- | --- | --- | --- | --- | --- | --- |
| Perception of the importance of the intervention. | Important, as it allows you to diagnose much faster. Can be treated faster, targeting opportunistic infections. And sometimes, it even manages to avoid hospitalizations, when it is in a more outpatient setting. Or treat faster in a hospital context, especially in hospitals with fewer resources, where there are no other gold standard tests. | Important because it allows early diagnosis of potentially lethal opportunistic infections, the diagnosis of which is usually difficult by traditional means. | It makes all the difference, because the investigation of these cases usually takes a long time, right? Generally, it is not the first attempt at investigation that a diagnosis is reached. | Fundamental because it provides a quick diagnosis of diseases, which would require time and invasive exams to be diagnosed and especially because it excludes diseases that would remain as diagnostic hypotheses.  Especially, histoplasmosis because it is a disease that mimics many others | Fundamental. | Testing faster, treats faster. We can minimize sequelae resulting from the infection. rapid diagnostic intervention allows for more targeted management in earlier stages of difficult-to-diagnose diseases. |  |
| Difficulties. | In hospital, the delay in carrying out the tests (72 hours and not 24 hours); interpretation difficulties, and another diagnostic resource to confirm the result. LAM test | Test performance, especially LAM test, because the interpretation is visual, or when there is false positive data. |  | LAM test. That many times we see a positive result and, in fact, the patient has another non-tuberculous mycobacterium |  |  | Patient adherence to wanting to take the tests, especially the most seriously ill patient. He's been stung a lot, he's been tested a lot, so he's always a little resistant. |
| What was your experience with rapid diagnostic intervention?  +  Questions and reactions from patients. | Collaboration with medical colleagues, cool to participate in a clinical study, difficulty in doubting confidence in the diagnosis. In research: every now and then we include a patient. Then, when I was going to test the patient, he was no longer hospitalized. | Suspicion about the LAM test, but as for the others, I didn't see any specific comments. It's just. | I think one of the facilitators was the fact that we won the tests. And the fact that we received training. At hospital there is no research history, so patients are sometimes surprised; Often, the patient is unable to consent. Because he is a patient, in short, he has a history of drug addiction, a history of a psychiatric illness. | Difficulties in coordinating the routine, because I end up doing the tests when the team signals it, we have a dedicated room for that, so. Certainly in an outpatient routine it must be very different. | It greatly facilitates the structure of the hospital, the central laboratory. Then, we can do the tests using pipettes, we don't need to lance the patients' fingers;  It's difficult to reconcile this with the hospital’s routine. The nurses have other things to do, so we often have to visit the patient 2 or 3 times until we can collect the sample. | Generally calm; We had some patient situations, for example, where both the patient and the patient's husband were illiterate, so this created a little more difficulty in explaining; The family also knew that the patient wanted to do the tests and the family did not want him to do the tests. So this created a conflict between the patient's wishes and the family's wishes. | Gratifying |
| How rapid diagnostic intervention influenced your clinical decision-making or relationship with the patient. | I'm more in infection control, I don't provide direct assistance. Assistant doctors who are clinicians have the most contact and they deal with patients' reactions after a positive or negative result; but I think it has a lot of potential to… to improve relationships with patients.  In this hospital, many patients are hospitalized without even understanding their health condition, without even knowing what HIV is, what it causes if it is not treated. When you explain the consent form and the objective of the research, you are also talking about the current situation. It's an enlightening conversation, perhaps much better and more complete than what he had up until that point with his attending physician (This impacted adherence to treatment, in the professional's view) |  | I'm an assistant doctor, right? So it didn't impact mine. In my therapeutic approach itself, but I think it helped in early diagnosis and in diagnosing situations that were previously undiagnosed |  | I am not included in the decision-making, the clinical part. | I am not included in the decision-making, the clinical part. | Even for them to also understand that it's not just HIV, which is the case with HIV, non-adherence to treatment, there may be more hidden things, right? |
| Improvements to be made in the hospitals where you work. | Difficulty in requesting these tests.  Some difficulty in terms of logistics as it is not a test that is in the system. It's not in the medical record.  In the civil approach, some thus need to call psychology to be together. And I think that the difficulties of Interdisciplinary (difficulties in accessing patients in research) | Leaving the context of the research, if the test was part of the routine, it would be much simpler, because the doctor prescribes it, the nurses go there and apply it via digital puncture, it could be directly the test, urine collection, the result comes out in the system, on the same day. In essential practice, I think it tends to work very well… | In hospital. A lot of work has been done in a multidisciplinary way to conduct the project. I think that for the project itself, the biggest difficulty is reconciling research activities with other activities; the multi team was essential to, for the project. |  |  | multi team was fundamental, mainly nurses and nursing technicians. But, due to the conditions of the patients, probes are often necessary. Yeah, or bedridden patients in the ICU.  Difficulties, being able to introduce this into something that is not in the routine. |  |
| Challenges faced when using rapid diagnostic intervention. | For expansion is the training of laboratory analysts. So if this became routine, everyone would have to be trained, it's not difficult, they are tests that are easy to interpret and execute.  Difficulties? I think it's being out of the routine even because it interferes with the already established work demand of nursing and laboratory professionals. Cost issue and that's it, in general. | They are minimal in terms of implementation because it is a non-invasive test. And, and fast. And easy to interpret. But the prospect of expansion involves cost, especially when it comes to SUS. And we need to have clearer cost-effectiveness studies to get an idea of the benefit of tracking these diseases or diagnosing them earlier to convince the administrator and reimbursement from insurance plans, especially in private hospitals.  Me. I think the benefit of testing asymptomatic people hospitalized with advanced AIDS is not clear. Not for cryptococcosis, that is already well defined, but perhaps for LAM test or for, for the histoplasma test . Or for those who have it, those who don't have AIDS but have an extremely high CD4 count, perhaps. The pre-test probability of disease is so low that it is not worth testing.  (risk of false positive + aggressive treatment vs Professional 1 adds: the benefits of a universal testing approach outweigh the risks) | Challenge was more related to research methodology. The request is made differently, not through the system, more related to research, not through the exam itself. Because the exams themselves are super easy to take.  And in relation to the expansion perspective, it is more related to financial resources, right? People management resource to carry out this implementation, and no, not the exam itself, but in relation to resource management. |  |  |  |  |

**Focus Group 2**

| Profile | P1 - 33 years old, I am also, I am in the third year of my residency at the hospital. And I've been treating patients with HIV AIDS for the last 3 years. | P2 - infectious disease specialist. Yes, I'm a professor of infectious diseases. And I am a principal investigator on several projects. Researcher in the area of HIV and serving people for, I don't know, 20 years, more or less | P3 - 28 years old I am now a final year resident in infectious diseases at the hospital. worked with HIV AIDS and these opportunistic infections for at least 3 years formally in residence |
| --- | --- | --- | --- |
| Perception of the importance of the intervention. | Patient survival by being able to quickly diagnose potentially serious illnesses and treat them quickly. | An antigen test that has good specificity and sensitivity with a positive result, confirming this hypothesis, brought us a lot of security (as a tool for confirming suspicion; but between the lines there still seems to be doubt about the accuracy and specificity of the test). ; What we hope is that it will actually reduce the morbidity and mortality of these people with advanced HIV, expanding the diagnostic capacity. | Essential in a context where these diagnoses were not widespread, they often took longer than a month to be processed. Time is crucial when it comes to treating these patients, because they are diagnosed with serious illnesses, with potential progression to adverse outcomes, such as death. Early diagnosis, initial treatment, this changes the chain of intervention and provides real access, the possibility of a cure. |
| Difficulties. | The patient understands a diagnosis of diseases that are so difficult to treat. And they thought that because it was discovered so easily, so quickly, everything was fine (difficulty adhering to treatment); Most of these diseases involve long treatments.  The specificity and sensitivity of the test. |  | Difficulties when we dealt with patients who were not ours, through consultancy or who were included through other means than ours directly in the research, we often dealt with some other professionals, mainly doctors, who did not initially believe in the potential, in the effectiveness diagnostic of the test, in the parameters of accuracy, sensitivity, specificity. |
| What was your experience with rapid diagnostic intervention? | For the most part, I had no problems. | The people understood it correctly and understood how to execute it. And we managed to execute it, which required virtually no infrastructure, other than a sink and, obviously, the material there for execution. It was very peaceful to do the tests here. | In general it was very easy. During hospital admission, the patient is often in a fragile health condition and any therapeutic option - diagnostic - is received very well, as a hope of improvement, of a quicker hospital discharge. So I think this was well received by patients.  (in the research) We had 2 or 3 situations involving consulting patients with other teams that were like “ah, what is this story? You are saying that my patient has this, but where did it come from?” But it was basically explaining the protocol, the tests and people understood |
| Questions and reactions from patients. | (difficulty) - when we approach the patient to explain what we are going to do, we say “ah, our objective is rapid tests for the 3 diseases that could be worse for you”. So, we try to bring it this way, but sometimes, how we deal with people who have a social issue - sometimes even education that is not so high. |  | The vast majority were patients who had already been diagnosed with HIV for perhaps many years. They were already in a context of treatment abandonment. So these diseases - tuberculosis, cryptococcosis - were not necessarily new to most. Some had already undergone these treatments previously. Of course, there were some patients who were diagnosed with HIV with these opportunistic infections, which, well, it was really the work of explaining that perhaps the treatment would be prolonged and everything that came with it, but for the vast majority it was more of a hospitalization. |
| Regarding the results obtained in the intervention, you are confident. |  | I think so, right? For everything that was said. Ease of execution, right? No need for laboratory infrastructure, greater speed. And the fact that we execute it ourselves or that people remarkably close to the team execute it. They are tests, like any diagnostic test, they have their characteristics, their sensitivity and specificity. But these are already validated tests. In the literature, there is already a solid basis for the use of these tests in PLWHA. What we are validating is the package of diagnostic interventions. This is what it means for these people living with HIV to offer these 3 tests together at this time. We will interpret the results on a case-by-case basis, but we have a lot of confidence in the tests. |  |
| How rapid diagnostic intervention influenced your clinical decision-making and relationship with the patient. | Most of our tests were done on patients who were already hospitalized. And then, as a result of their diagnoses, they had a hospital stay. From what I remember of ours to this day. And I think the main influence on our decision will be whether or not to start treatment, right? For the diagnosis that the test will tell us or not. | The results of the study sometimes ended up motivating the patient to be hospitalized for treatment. So this was a consequence that happened in some patients. Likewise, additional investigation, right? So, a patient who is sometimes well, we would not decide on hospitalization, but especially in the case of histoplasmosis, we decide to investigate further to see, eventually, if he has any signs of hidden disease that we can detect with further investigation. So, this kind of situation also happened. | With these diagnoses, with these tests, it has now been possible for us to speed up a process that is not so long, but which sometimes takes a few days, and for the patient to receive a treatment that has fewer adverse effects more quickly. |
| Interdisciplinary Collaboration. | We didn't lose one or two of the segments because they escaped, they left. They didn't even know what the outcome of their evaluation, their treatment, the way they were, right? Even at the beginning, we were asking the nursing staff and such to collect the samples. Now, in recent times, we go there and collect and do our own collection and it worked. | Research is one thing, right? In the research, in our time here, basically, it was the medical team that did it. With some very punctual intersections. But in other centers it was different.  My interaction with professionals on the project was minimal. Eventually some difficulties occurred, there were some samples that were lost, we had to collect them again, but they were isolated things. In general, it went well.  Unfortunately, they occur a lot in patients with this profile with advanced HIV. Most of the patients there at the hospital were abandonment patients, they had lost segment. With this patient profile, if we want to test whether an intervention will actually impact morbidity and mortality, ideally, it would be nice if we had a truly multidisciplinary intervention. So, if we had a segment of these people, with nurses, social workers, which are many patients who sometimes have important vulnerabilities, right? The pharmacist himself to take care of. So, it is something quite common in this population, so, of course, it is outside the scope of the project. But a multidisciplinary intervention would benefit these people. | Mainly doctors. We had help from nursing at first. We needed a blood sample, a urine sample, but we realized that after… well, as time passed, there were more… several little problems.  He collected a blood sample, did not find the resident, so the blood clotted. And then, well, the problems started happening. The pee that was collected for the research was not sent to the laboratory unintentionally. He arrived at the laboratory, the laboratory did not understand why pee arrived in the laboratory. So after that we started to resolve things ourselves so that we could be more in control of the flow.  And it is important to say that this was very specific to the research, the application of the tests, because if we are going to talk about hospitalization for advanced AIDS, we have the multidisciplinary team in all gaps and all spaces, from the pharmaceutical team, from the nursing. |
| Recommendations. | How will this diagnostic package be carried on the basic network? There will have to be training, something. For example, a recently graduated doctor who is working in a post somewhere, in a UBS and is going to receive a patient who is from his region. And what will be done about it? If we are going to make this diagnosis, we will have to have a hospital back-up, and perhaps we will need to admit these patients, carry out additional investigation and even what will be plausible about this or not? So, I think there is another bureaucratic organizational part that still needs to be considered in relation to this. | It's impossible for us to simply play this test today. The network cannot even absorb HIV patient care well. HIV without severe immunosuppression, HIV that is more controlled, is already difficult, it is no longer possible. Imagine advanced HIV. So, I think this is a challenge that will remain. But I think our role here within the study and what we are trying to fulfill is to inform the scientific community with evidence. |  |
| Challenges faced when using rapid diagnostic intervention. |  | The patient profile, right? So, we can actually reach all patients who would be eligible. We have a lot of people who are lost in the flows, with advanced HIV, with the disease progressing and who are not having access to hospitals and who are in the program, so I think that for me, from a macro point of view, this is the biggest challenge. We had internal challenges, in relation to teams, collections, but in my understanding these are operational challenges that we will always have in any study. I think the macro challenge here on the HIV issue is to expand access.  In expansion, we paint this picture for managers so that the decision can then be taken by more, with more reliable data, right? What's going on? Who are these people? What does the test impact or not, and based on that, whether it is worth implementing or not. | These are neglected diseases, so this negligence is not only conceptual, but we know that it comes from the lower number of public incentives, the greater difficulty in accessing these patients, the social layers that these patients in general are representing. So we understand that there is an access challenge, there is underreporting, even though these tests make it easier for us from the point of view of making a faster diagnosis, of introducing faster therapy. |
