# Supplementary material for: Experiences and perspectives on rapid-test diagnosis of tuberculosis, histoplasmosis and cryptococcosis in people with advanced HIV/AIDS disease in Porto Alegre, Brazil
Source: PLoS One. 2024 Nov 27;19(11):e0312204. doi: 10.1371/journal.pone.0312204 (PMC11602015; doi:10.1371/journal.pone.0312204)
Supplement: S4 Appendix — (DOCX) [file pone.0312204.s004.docx]

**Appendix S4**

**Summary table of interviews with patients**

| Profile | P1- Female, straight, divorced, has children, class C, capital, completed primary school, white. | P2 - 58 years old, male, straight, married, has children, class C, rural, incomplete primary education, white. | P3 - 34 years old, female, straight, single EU, has children, class, capital,  incomplete elementary school, black. | P4 | P5 - 44 years old, female, straight, single EU, has children, class E, capital, incomplete elementary school, mixed race. | P6 | P7 - Male, full upper, white. | P8 - 37 years old, male, married, straight, has children, class D, capital, incomplete elementary school, mixed race. | P9 - | P10 - 37 years old, male, homosexual, single EU, no children. no income, completed high school, white. | P11 - 60 years old male, straight, divorced, has children, class D, capital, completed primary school, white. | P12 – 38 years old, male, straight, married, no children, class E, capital, completed primary school, mixed race. |
| --- | --- | --- | --- | --- | --- | --- | --- | --- | --- | --- | --- | --- |
| Diagnosis time and conditions | Approx. 10 years. | Less than a year. | 11 years. I was going to have surgery to remove the tube in the hospital. He wasn't being treated. | 27 years. He doesn't know how he got it. Friend suggested testing because they were doing testing while she was taking care of her grandmother in the hospital. | 16 years. The partner had it, so he tested it. | 20 years. A companion had it. Then, he found out he had it, saw an advertisement and decided to take the test to confirm. He started treatment 5 years ago. | 13 years. | This year he discovered in an exam that he had to pull out a tooth. Doctors reported that he has probably been living with the virus for more than 10 years. | At 12 years old. (no age information). She was hospitalized with low immunity. Transmission occurred from mother to daughter, it seems that she was born with the virus. | 2-3 years. He got sick, lost weight, and was diagnosed at the hospital. | nine years old. Exams due to cancer. | 15 years. He got it from his first wife. She found out why she tested trying to get pregnant, he took the test soon after. |
| Opportunistic diseases | Maybe histoplasmosis. The others believe not.  Chickenpox. | None.  My stepson had tuberculosis. | Tuberculosis . “The tuberculosis I had is the one that doesn’t spread to anyone, it’s from the lungs” | No, you don't even know anyone who has had it.  Toxoplasmosis. | No, it never was. She had tests done when she was hospitalized, she doesn't remember.  An acquaintance had tuberculosis and sought help at the hospital.  Meningitis. | Histoplasmosis. | Tuberculosis. Diagnosed without symptoms in a routine exam. | No. | No.  Tuberculosis was suspected , but it was negative.  He doesn't know anyone who has had it.  HPV.  Pneumonia. | No.  Pneumonia. | Tuberculosis.  Meningitis. | Tuberculous. |
| Difficulties  And expectations about the intervention | Not with the service itself.  Health difficulties (convulsions, starvation).  wasn't treated for many years, it's... Immunity was extremely low | I didn't worry about that, exams. (the participant focuses his answers on the impact of discovering he is HIV positive and leaves aside the issue of rapid testing) | Not being able to walk, not being able to see your children The hospital does not allow children to visit, lung pain. (patient reported difficulties, but not about testing) | No. | - | He doesn't remember when he took the exam. | “the biggest problem related to patients with HIV is the lack of respect from healthcare teams” (again, a patient responds focused on the HIV/ AIDs experience , not the test) | No. | - | - |  | Not about the exam, about the treatment, difficulty adapting to the medicine and being able to eat. |
| Did the rapid diagnosis take time? | No | I didn't know, I found out later. | One week after arrival at the hospital. | - | - | Don't remember. | Is unaware of the intervention. | No. | Yes. 2-3 days | - | - | Yes. (Saturday to a Sunday) |
| Symptoms and time from first symptom to diagnosis | Two days at the health center.  .  Approx. 50 days hospitalized | - | Pumping out blood, lung pain, not walking, organ damage.  Two weeks at the health center, several tests, but for tuberculosis only at the hospital. In the hospital, a week until being informed of the diagnosis, 2-3 months hospitalized. | Paralytic (toxoplasmosis). | Intracranial pressure, vomiting, headache; I couldn't open my eyes, move or get up (meningitis).  In the health center, a week in the emergency room, then in the hospital. It was already unbelievably bad before I went to the clinic, I don't know how long it was. | Psychotic break, doesn't remember some things. He stayed at hospital . Before that, the first place she was taken was a polyclinic psychiatric clinic in Ulbra (four sleepless nights, visual hallucinations)  It took two months to stay in hospital. | No symptoms. | - | “Symptoms of a flu ”  5 days until you have all the test results. It had been bad for a few days. After having the flu, which developed into pneumonia, he was at home self-medicating. | “shortness of breath, a lot of pain in my back, a lot of pain in my chest” (pneumonia)  3 months.  He went to three hospitals before being diagnosed in hospital (I think he is referring to the AIDS/HIV diagnosis here) | “very severe headache”  He went to UPA, went to hospital, had a high, and got worse at home. It was at the hospital, where he was hospitalized for 30-40 days. In the hospital he caught Covid, spending another 20-30 days in hospital. | He had no symptoms, they did tests because he was thin.  21 days admitted to hospital. |
| Clear and sufficient information about the intervention | Yes | Yes | Yes, but he didn't receive, nor was he able to get the results of the tomography. | No. | Yes, but they spoke to her children, relatives and daughter-in-law, she was unconscious in the ICU , unresponsive (meningitis).  Regarding the three diseases, he says he took tests at the health center, but it was always negative. | The doctors spoke to the children. | Yes, you received the results. | Yes. | Yes. | Yes. (I believe the answers refer to the HIV/AIDS diagnosis) | Yes. | Yes. |
| Influence on treatment and quality of life, benefits | Being able to treat the disease so you don't... so you don't end up getting other, worse diseases. | Monitoring, assistance, care. Every consultation you make , you wait to hear something good, a retraction | I don't know. | I don't know. “I no longer have any expectations, I live today, because I don't know if I'll be here tomorrow.” | “Knowing if everything is ok or not, I think the only benefit is that” | “It’s good, because then it’s faster to fight” | PLWHA should have a faster flow, as soon as they are diagnosed, without “popping up” | - | The faster the result, the faster the concern can be resolved (if the result is negative) or progress towards treatment. | - | - | Refer for treatment. |
| Difficulty accessing, performing or receiving results | No | Treatment could be more isolated, the biggest barrier is shame... | No. | No.  “For me, there are no longer any barriers to the type of life I’m living, okay?” | No. | I wouldn't know how to answer you. | - | - | No. | No. (reported allergy/initial reaction to HIV/AIDS treatment) | No.  (I miss walking, I miss a quicker diagnosis, like this...  Until shortly before I [...] I worked normally. I had my movements, because my profession is a truck driver). | No. Fast and agile. |
| Concerns about the intervention | None | None | No. | I don't know. | - | - | - | Someone you know see. Gossip. | With the family. (how will she receive the result) | - | - | No. |
| Difficulties implementing intervention in the health service | I can't say. | - | - | I don't know. | No. | - | - | - | - | No obstacles.  As “a somewhat ignorant person when it comes to this kind of thing, I don’t like hospitals, I’m afraid of needles, things like that and people” rapid diagnosis helps, plus welcoming | “Very well treated, very well attended. There's no... There's nothing bad, there's no way to get any better, no. Even more for SUS” | "No. From the beginning of the hospital to the end of [...] CAPS and the health center, it's a... It's a group that worked together with us, together with the patient, understand? It went really well. It was wonderful." |
| Recommendations to improve intervention | The way it is being done, everything is fine | The Brazilian health system is not highly effective, but the number of doctors who work there is good. But they don't have what we can do anymore , you know, there's nothing else to do. | No. I don't know. I was treated well in the hospital.  “What a pill. Could there be an injection? Oh, it's my dream.” (refers to HIV/AIDS treatment) | I would trust more in professionals specialized in this part of, well, STIs or infections. Doctors, nurses, everything related to healthcare.  God. It would be my grandchildren, my children, my husband, my mother, my mother-in-law. (about improving quality of life for PLWHA) | I don’t know, but “there should be more places, more health centers than people. Take the exams, take the medications. Not in a single place like there... At the gas station in the health center.  This is difficult for me.” | From what I remember. “I have always been treated well”  Where would you prefer to be tested? suddenly in the house? “Look, the way I feel today, I wish it were at home. I have such a trauma that I don't want to go out on the street. For me it would be great.” | Regardless of the tests, the first improvement is the qualification of professionals to provide better care, with more respect for patients, with more agility and with more accurate information regarding referrals. There is no point in having a diagnosis if there is no appropriate treatment. | I was well received, it was great. I trust nurses, doctors.  “it had to be more of a psychologist.' (most of the patient's responses are about the recent discovery of HIV/AIDS, in this case, for example, he talks about the suffering of discovering the diagnosis and the importance of the psychologist). | I believe that there could also be smaller clinics, other health units.  Without a predilection for professionals, “our system is very overloaded” “If this were more easily available in any position, it would be enough”. | “I was well taken care of, understand? So I got there to a minimum expectation and left there well. I left there wanting to get treatment” (HIV/IDS diagnosis, not about this)  BUT  “Home care also from staff at the station, mine, the community where I live. | “I had, I wanted there to be a more effective medicine, you know. A more effective treatment for this disease. And that it wouldn’t get in the way of my daily life.”  “I don’t know if it’s necessary to take as much medicine as I do. I take, I take about 15 pills” | “they are doing a very good job” “this part of the rapid test, that's it, I was impressive, impressed to see the rapid test there that he did in front of me, with the nurse at the station. It went really well. So now I feel fulfilled and very, very happy? For me it’s great, it’s a 10 for them.” |
