# Supplementary material for: Experiences and perspectives on rapid-test diagnosis of tuberculosis, histoplasmosis and cryptococcosis in people with advanced HIV/AIDS disease in Porto Alegre, Brazil
Source: PLoS One. 2024 Nov 27;19(11):e0312204. doi: 10.1371/journal.pone.0312204 (PMC11602015; doi:10.1371/journal.pone.0312204)
Supplement: Supplementary file 4 [file pone.0312204.s002.docx]

Appendix S2

General characteristics of patients

Gender identity	Gender assigned at birth	Sexual orientation	Education	Occupation	Race	Monthly family income (R$)	Year of HIV diagnosis
CIS man	Masculine	Heterosexual	Middle School	Construction worker	White	1750	2010
CIS man	Masculine	Heterosexual	Middle School	Taxi driver	White	2000	2023
CIS Woman	Feminine	Heterosexual	Middle School	Homemaker	White	No income	2023
CIS Woman	Feminine	Heterosexual	Middle School	Day laborer	White	1300	2000
CIS man	Feminine	Heterosexual	High School	Factory salesperson	White	1500	2007
CIS man	Masculine	Homosexual	High School	Event promoter	White	2800	2016
CIS man	Masculine	Heterosexual	Middle School	Truck Driver	White	2000	2014
CIS Woman	Feminine	Heterosexual	Middle School	Unemployed	Black	2204	2012
CIS Woman	Feminine	Heterosexual	Middle School	Unemployed	Black	800	2009
CIS man	Masculine	Heterosexual	Middle School	Mechanic	White	2035	2023
CIS man	Masculine	Heterosexual	High School	Gardening assistant	Brown	1200	2023
CIS Woman	Feminine	Heterosexual	Middle School	Housekeeper	White	3000	2011
