# Supplementary material for: Experiences and perspectives on rapid-test diagnosis of tuberculosis, histoplasmosis and cryptococcosis in people with advanced HIV/AIDS disease in Porto Alegre, Brazil
Source: PLoS One. 2024 Nov 27;19(11):e0312204. doi: 10.1371/journal.pone.0312204 (PMC11602015; doi:10.1371/journal.pone.0312204)
Supplement: Attachment — Submitted filename: Response to Reviewers.docx [file pone.0312204.s005.docx]

**Response to Reviewers**

Title manuscript: **Experiences and perspectives on rapid-test diagnosis of tuberculosis, histoplasmosis and cryptococcosis in people with advanced HIV/AIDS disease in Porto Alegre, Brazil**

**Ref: Submission: PONE-D-24-25548**

**Editorial Comments**

The study presents a methodology appropriate to the objectives and the manuscript is well written, although revision is necessary,

as in line 462: a) ....... (infect ologists).

Its results provide a contribution to the implementation of a screening and treatment strategy recommended by the WHO in a Brazilian metropolis, however, some aspects could be better explored in the discussion as healthcare workers: training of health professionals and multidisciplinarity of the health team; as policemakers: necessary human resources and formation of a care network (primary, secondary), regulation of the health system, surveillance and management of the system, including the demand of patients for specialized professionals (infectologists) and formulation of care and treatment protocols, as well as interfederative collaboration.

Please clarify how the random selection of participants (patients) for the interview was carried out?

1. When submitting your revision, we need you to address these additional requirements.

**Please refer to the responses related to these matters below**

Please ensure that your manuscript meets PLOS ONE's style requirements, including those for file naming. The PLOS ONE style templates can be found at

https://journals.plos.org/plosone/s/file?id=wjVg/PLOSOne_formatting_sample_main_body.pdf and

<https://journals.plos.org/plosone/s/file?id=ba62/PLOSOne_formatting_sample_title_authors_affiliations.pdf>.

**This has been done**

2. We note that the grant information you provided in the ‘Funding Information’ and ‘Financial Disclosure’ sections do not match.

When you resubmit, please ensure that you provide the correct grant numbers for the awards you received for your study in the ‘Funding Information’ section.

**We will take this in consideration in the resubmission**

3. We note that you have indicated that there are restrictions to data sharing for this study. PLOS only allows data to be available upon request if there are legal or ethical restrictions on sharing data publicly. For more information on unacceptable data access restrictions, please see http://journals.plos.org/plosone/s/data-availability#loc-unacceptable-data-access-restrictions.

Before we proceed with your manuscript, please address the following prompts:

a) If there are ethical or legal restrictions on sharing a de-identified data set, please explain them in detail (e.g., data contain potentially identifying or sensitive patient information, data are owned by a third-party organization, etc.) and who has imposed them (e.g., a Research Ethics Committee or Institutional Review Board, etc.). Please also provide contact information for a data access committee, ethics committee, or other institutional body to which data requests may be sent.

**Interview and focus group data cannot be fully disclosed due to the nature of the consent signed by participants, which did not include the provision for fully anonymized transcripts to be made available. Given the limited number of health professionals and public managers involved in the topics discussed in this manuscript in Porto Alegre, anonymization efforts would still not prevent the identification of participants if the full transcripts were released. This would risk violating the data privacy requirements set by the university research ethics committee (PUCRS). Data requests may be directed to the Pontifical Catholic University of Rio Grande do Sul University (Pontificia Universidade Católica do Rio Grande do Sul) Ethics Committee at cep@pucrs.br**

b) If there are no restrictions, please upload the minimal anonymized data set necessary to replicate your study findings to a stable, public repository and provide us with the relevant URLs, DOIs, or accession numbers. For a list of recommended repositories, please see

https://journals.plos.org/plosone/s/recommended-repositories. You also have the option of uploading the data as Supporting Information files, but we would recommend depositing data directly to a data repository if possible.

We will update your Data Availability statement on your behalf to reflect the information you provide.

4. In the online submission form, you indicated that [The data underlying the results presented in the study are available from request of the correspondent author.].

All PLOS journals now require all data underlying the findings described in their manuscript to be freely available to other researchers, either 1. In a public repository, 2. Within the manuscript itself, or 3. Uploaded as supplementary information.

This policy applies to all data except where public deposition would breach compliance with the protocol approved by your research ethics board. If your data cannot be made publicly available for ethical or legal reasons (e.g., public availability would compromise patient privacy), please explain your reasons on resubmission and your exemption request will be escalated for approval.

**Interview and focus group data cannot be fully disclosed due to the nature of the consent signed by participants, which did not include the provision for fully anonymized transcripts to be made available. Given the limited number of health professionals and public managers involved in the topics discussed in this manuscript in Porto Alegre, anonymization efforts would still not prevent the identification of participants if the full transcripts were released. This would risk violating the data privacy requirements set by the university research ethics committee (PUCRS). Data requests may be directed to the Pontifical Catholic University of Rio Grande do Sul University (Pontificia Universidade Católica do Rio Grande do Sul) Ethics Committee**

**at cep@pucrs.br**

5. Please include captions for your Supporting Information files at the end of your manuscript, and update any in-text citations to match accordingly. Please see our Supporting Information guidelines for more information: http://journals.plos.org/plosone/s/supporting-information.

**This has been considered in the manuscript.**

**Additional Editor Comments:**

The study presents a methodology appropriate to the objectives and the manuscript is well written, although

revision is necessary, as in line 462: a) ....... (infec tologists). Its results provide a contribution to the implementation of a screening and treatment strategy recommended by the WHO in a Brazilian metropolis, however, some aspects could be better explored in the discussion as healthcare workers: training of health professionals and multidisciplinarity of the health team;

**We thank the editor for this suggestion.** **We have included a paragraph based on this suggestion (page 15-16, line 474-484)**

as policemakers: necessary human resources and formation of a care network (primary, secondary), regulation of the health system, surveillance and management of the system, including the demand of patients for specialized professionals (infectologists) and formulation of care and treatment protocols, as well as interfederative collaboration.

**Here again, we thank the editor for this suggestion. We have included a paragraph based on this suggestion (page 15-16, line 474-484)**

Please clarify how the random selection of participants (patients) for the interview was carried out?

**This has been clarified, we have added a sentence (page 5, line 143-146)**

**Reviewers' comments:**

Reviewer's Responses to Questions

Comments to the Author

1. Is the manuscript technically sound, and do the data support the conclusions?

The manuscript must describe a technically sound piece of scientific research with data that supports the conclusions. Experiments must have been conducted rigorously, with appropriate controls, replication, and sample sizes. The conclusions must be drawn appropriately based on the data presented.

Reviewer #1: Partly

Reviewer #2: Yes

________________________________________

2. Has the statistical analysis been performed appropriately and rigorously?

Reviewer #1: I Don't Know

Reviewer #2: Yes

________________________________________

3. Have the authors made all data underlying the findings in their manuscript fully available?

The PLOS Data policy requires authors to make all data underlying the findings described in their manuscript fully available without restriction, with rare exception (please refer to the Data Availability Statement in the manuscript PDF file). The data should be provided as part of the manuscript or its supporting information, or deposited to a public repository. For example, in addition to summary statistics, the data points behind means, medians and variance measures should be available. If there are restrictions on publicly sharing data—e.g. participant privacy or use of data from a third party—those must be specified.

Reviewer #1: Yes

Reviewer #2: Yes

________________________________________

4. Is the manuscript presented in an intelligible fashion and written in standard English?

PLOS ONE does not copyedit accepted manuscripts, so the language in submitted articles must be clear, correct, and unambiguous. Any typographical or grammatical errors should be corrected at revision, so please note any specific errors here.

Reviewer #1: Yes

Reviewer #2: Yes

________________________________________

5. Review Comments to the Author

Please use the space provided to explain your answers to the questions above. You may also include additional comments for the author, including concerns about dual publication, research ethics, or publication ethics. (Please upload your review as an attachment if it exceeds 20,000 characters)

**Reviewer #1**:

Clearly describe what type of secure software was used to store the survey data.

**This has been described, page 5, line 124-126**

Describe the cost of implementing the diagnostic package.

**This has been added in the manuscript with the corresponding references, page 15, line 459-462**

Present and describe proposals to intensify adherence to the proposed intervention based on the difficulties highlighted in the study.

**We thank the reviewer for this suggestion. We have added a paragraph on this, page 17, line 528-538**

**Reviewer #2**:

The study was well conducted from a scientific standpoint, and the manuscript is very well written. Its results provide a significant contribution to understanding how the implementation of a 'screen and treat' strategy recommended by the WHO was perceived by patients, healthcare professionals, and managers in a Brazilian metropolis. This knowledge, which goes beyond the accuracy of the tests used, offers crucial scientific support for improving the implementation of this strategy, considered a priority in reducing AIDS-related deaths worldwide.

I have three suggestions to improve the manuscript:

1. In the Methods section:

a. To help the reader better understand the perceptions of patients, healthcare professionals, and managers regarding the strategy of screening for opportunistic infections with rapid tests (rapid diagnostic intervention), the authors should provide a detailed description of how this strategy was carried out. This includes which tests were used, how long it took, how the tests were conducted, and by whom.

**We thank the reviewer for this suggestion to improve the manuscript. We have clarified this with a statement, page 3, line 79-87**

b. The authors should clarify in the methods section from which settings the patients and healthcare professionals were selected, just as they did when describing the selection of the managers.

**This was clarified in page 4, line 105-106 and 110-111**

2. In the Discussion section: Healthcare professionals expressed concerns about the accuracy of the tests, which could negatively impact the implementation of the strategy. I suggest that the authors discuss possible measures to minimize this issue in the paragraph on line 429.

**We thank the reviewer for this suggestion, we have added a statement in page 15, line 452-454**
